# Automated noninvasive detection of idiopathic scoliosis in children and adolescents: A principle validation study

**DOI:** 10.1038/s41598-018-36360-w

**Published:** 2018-12-07

**Authors:** Hideki Sudo, Terufumi Kokabu, Yuichiro Abe, Akira Iwata, Katsuhisa Yamada, Yoichi M. Ito, Norimasa Iwasaki, Satoshi Kanai

**Affiliations:** 1 Department of Advanced Medicine for Spine and Spinal Cord Disorders, Faculty of Medicine and Graduate of Medicine, Hokkaido University, N15W7, Sapporo, Hokkaido 060-8638 Japan; 2 Department of Orthopaedic Surgery, Hokkaido University Hospital, N14W5, Sapporo, Hokkaido 060-8648 Japan; 3 Department of Orthopaedic Surgery, Eniwa Hospital, Koganechuo 2-1-1, Eniwa, Hokkaido 061-1449 Japan; 4 Department of Biostatistics, Hokkaido University Graduate School of Medicine, Sapporo, Hokkaido Japan; 5 Division of Systems Science and Informatics, Hokkaido University Graduate School of Information Science and Technology, N14W9, Sapporo, Hokkaido 060-0814 Japan

## Abstract

Idiopathic scoliosis is the most common pediatric musculoskeletal disorder that causes a three-dimensional deformity of the spine. Early detection of this progressive aliment is essential. The aim of this study is to determine outcomes using a newly developed automated asymmetry-evaluation system for the surface of the human back using a three-dimensional depth sensor. Seventy-six human subjects suspected to have idiopathic scoliosis were included in this study. Outcome measures include patient demographics, radiographic measurements, and asymmetry indexes defined in the automated asymmetry-recognition system. The mean time from scanning to analysis was 1.5 seconds. For predicting idiopathic scoliosis of greater than 25°, the area under the curve was 0.96, sensitivity was 0.97, and specificity was 0.88. The coefficient of variation for repeatability analyses using phantom models was 1–4%. The intraclass correlation coefficient obtained for intra-observer repeatability for human subjects was 0.995. The system three-dimensionally scans multiple points on the back, enabling an automated evaluation of the back’s asymmetry in a few seconds. This study demonstrated discriminative ability in determining whether an examinee requires an additional x-ray to confirm diagnosis.

## Introduction

Idiopathic scoliosis (IS) is the most common pediatric musculoskeletal disorder that causes a three-dimensional (3D) deformity of the spine^1^. Radiography is the usual method for assessing the severity of scoliosis, monitoring its progression, and guiding treatment decisions^2^. The standard measurement for quantifying scoliosis deformity is the Cobb angle, which is the coronal plane angle measured between the vertebrae at the upper and lower limits of the curve^2,3^. The onset of IS typically coincides with the adolescent growth spurt. The curve in moderate scoliosis (25–40°) is treated nonoperatively, using a brace to prevent further progression. Severe IS (>40–50°) warrants surgical correction to prevent later disfigurement and other co-morbidities, including back pain and pulmonary dysfunction^4–6^.

As early detection of the progressive aliment is regarded as essential for treatment, and frequent observation is required to determine if scoliosis curvatures worsen during the growing years^7^. Mass school scoliosis screening (SSS) programs for early detection of IS were initiated in the 1950s^8^. However, the use of SSS remains controversial, due to its false positive referral rate and excessive costs^8,9^. In 2004, the United States Preventative Services Task Force recommended against routine screening of asymptomatic adolescents for IS^10,11^. However, the American Academy of Orthopaedic Surgeons, the Scoliosis Research Society, the Pediatric Orthopaedic Society of North America, and the American Academy of Pediatrics published a position statement expressing that the importance of educating screening personnel to optimize the appropriate use of spine radiographs, as not all children referred as a result of screening require radiographs^12^. If radiographs were needed, physicians were advised to take necessary precautions to limit the patient’s exposure to radiation^12^. The average patient had 24.7 radiographs during treatment, and the mean estimated cumulative radiation dose was 10.8 cGy, which leads to a 1.7-fold increase in the risk of dying of breast cancer for female patients^13,14^.

Several methods can be used to screen for IS. The most basic method of these is clinical examination in Adam’s forward bending test with the use of a scoliometer^15,16^; 3D ultrasound (US) imaging is a promising method with the advantages of using nonionizing radiation^17,18^. Although this examinations are highly sensitive (83.3%) and specific (86.8%) for Adam’s forward bending test^19^ and have a moderate linear correlation with Cobb’s x-ray methods (y = 1.1797, R^2^ > 0.76) for US imaging^18^, the examiner must decide where the scoliometer or the US imaging probe should be placed and measure manually—a process that requires considerable time—which places a burden on doctors or school nurses who must examine many students within a limited time fame.

Surface topography (ST) is a method of trunk shape evaluation based on external body contour assessment that can be performed with several techniques. The historical Moiré ST metric was based on the interference of grids optically projected onto the subject’s back^20–23^. However, this technique requires that the light-source direction be perpendicular to the back surface, which is not possible with forward bending of the trunk, resulting in false positive rates of 32–60%^20–23^. In addition, an examiner must manually decide whether an asymmetrical image is suspected IS. Thus, most trained medical observer or similar for most screening programs never adopted this method. Improvements to the current system are therefore highly warranted; an innovative system should be noninvasive and automatic, and produce highly reliable quantitative measurements compatible with the Cobb angle.

We have developed a new system that solves the difficult problem of identifying IS. The system can evaluate the degree of asymmetry on the surface of the back from 3D measurements, enabling the automated evaluation of the back’s symmetry in a few seconds. In this way, it enables speedy and accurate detection of IS. This system does not need special preparation procedures or external markers, eliminating the delays associated with capturing images of the back surface. This study determines the outcomes of the newly developed automated asymmetry-recognition system.

## Methods

### System Configuration

**Figure 1:**
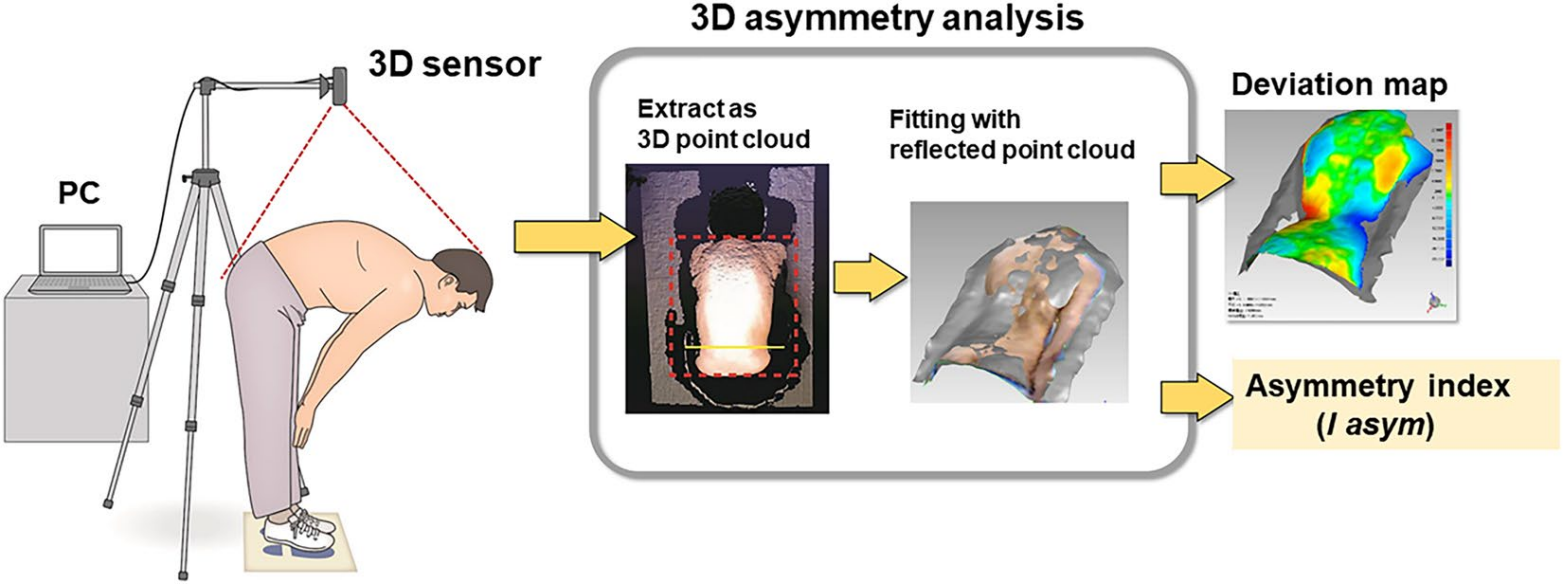
Experimental device and algorithm for the detection of 3D asymmetries. The device consists of a 3D scanner and a laptop computer. A subject bends forward and the surface of the back is captured by the scanner.

The system consists of a consumer-grade 3D depth sensor (Xtion Pro Live, ASUSTeK Computer Inc. Taipei, Republic of China) and a laptop computer (Core-i7, 7200U-16 GB CF-SZ5, Panasonic Inc, Tokyo, Japan). The depth sensor has depth measurement range of 0.8–3.5 m; horizontal, vertical, and diagonal view angles of 58°, 45°, and 70°; depth image sizes of 320 × 240 pixels (QVGA) and 640 × 480 pixels (VGA); and space sampling resolution at horizontal and vertical distances of 800 mm, 2.8 mm (QVGA), and 1.4 mm (VGA), and diagonal distances of 2.2 mm (QVGA and VGA). This system can be immediately used following installation of the external 3D deep sensor, which can be set up within a few minutes (Fig. 1). In addition, the sensor needs only a single calibration.

### Asymmetry Analysis Algorithm

An overview of our fully automated algorithm for detecting 3D asymmetry is shown in Fig. 1. Asymmetry analysis is performed and an asymmetry index ***I***_***asym***_ is calculated as follows:

#### Stage 1: Capture point clouds of a patient’s back

The surface of the patient’s back is scanned by the depth sensor so that the transverse lines of right and left of the waist are roughly aligned with the recommended lines displayed on the computer monitor. A 3D point cloud ${{\boldsymbol{P}}}^{0}(\,=\,\{{{\boldsymbol{p}}}_{{\boldsymbol{i}}}^{0}\})$ is captured that includes the back, breech, neck, and occipital surfaces and a floor, where ${{\boldsymbol{p}}}_{{\boldsymbol{i}}}^{0}$ denotes the 3D coordinates of a point included in point cloud ***P***^0^.

The original points in ***P***^0^ from the sensor typically include a non-negligible amount of errors and outliers. To reduce them, a smoothing process with a 3D moving average filter^24^ is first applied to ***P***^0^ to obtain the smoothed point cloud ***P***^1^, as shown in Supplementary Fig. 1A.

#### Stage 2: Segment the point cloud

Since the original point cloud ***P***^1^ still includes unnecessary points from the floor, ***P***^1^ is first partitioned into several connected point regions using a smoothness-constraint-based region growing algorithm^25^. As shown in Supplementary Fig. 1A, a point cloud ***P***^2^ that includes only the back, breech, neck, and occipital surfaces is extracted by taking the region closest to the scanner for which the sum of the side lengths of the region bounding box exceeds 650 mm.

#### Stage 3: Estimate a median sagittal plane

As shown in Supplementary Fig. 1B, principal component analysis is applied to ***P***^2^, and the pose-normalized point cloud ***P***^3^ is obtained from ***P***^2^ where the approximate median sagittal plane is aligned along the *x* and *z* axes of ***P***^3^.

#### Stage 4: Estimate the boundaries of the back surface

***P***^3^ still includes points on the breech, neck, and occipital surfaces—which are unnecessary for asymmetry analysis of the back surface and need to be removed. To this end, as shown in Supplementary Fig. 2A, P3 is first projected to its *xy* plane to generate a binary image ${{\boldsymbol{I}}}^{3}=$ $\{{\boldsymbol{b}}({\boldsymbol{i}},{\boldsymbol{j}})\,|{\boldsymbol{i}}=\frac{{\boldsymbol{x}}-{{\boldsymbol{x}}}_{{\boldsymbol{\min }}}}{{\rm{\Delta }}{\boldsymbol{x}}}\in \{0,{{\boldsymbol{i}}}_{{\boldsymbol{\max }}}\},{\boldsymbol{j}}=\frac{{\boldsymbol{y}}-{{\boldsymbol{y}}}_{{\boldsymbol{\min }}}}{{\rm{\Delta }}{\boldsymbol{y}}}\in \{0,{{\boldsymbol{j}}}_{{\boldsymbol{\max }}}\},{\boldsymbol{b}}\in \{0,1\}\}$ where a black pixel (***b***(***i***, ***j***) = 1) includes at least one projected point, and a white pixel (***b***(***i***, ***j***) = 0) does not. Δ***x*** and Δ***y*** denote pixel resolutions of the *x* and *y* direction, and ***x***_***min***_, ***x***_***max***_, ***y***_***min***_ and ***y***_***max***_ denote the ranges of *x, y* coordinates of the points in ***P***^3^. $\lfloor x\rfloor$ denotes a floor function that gives the largest integer that does not exceed ***x***. Then, the width of the black pixel region along the *y* direction at each discrete *x* position in ***I***^3^ is evaluated as the discrete width function

$${{\boldsymbol{w}}}_{{\boldsymbol{d}}}({\boldsymbol{x}})=\sum _{{\boldsymbol{j}}\in \{0,{{\boldsymbol{j}}}_{{\boldsymbol{\max }}}\}}\,{\boldsymbol{b}}(\lfloor ({\boldsymbol{x}}-{{\boldsymbol{x}}}_{{\boldsymbol{\min }}})/{\rm{\Delta }}{\boldsymbol{x}}\rfloor ,{\boldsymbol{j}}).$$

Finally, a high-order polynomial ***w***_***y***_(***x***) is fit to ***w***_***d***_(***x***) between ***x***_***min***_ and ***x***_***max***_. We adopted the 10^th^-order polynomial as ***w***_***y***_(***x***), which gives appropriate results. Next, by taking some local minima of polynomial ***w***_***y***_(***x***), the boundaries between the back and neck and the back and breech can be identified, and a rectangular region ***I***^4^ is extracted from ***I***^3^ that excludes the breech, neck, and occipital surfaces and is suitable for asymmetry analysis (Supplementary Fig. 2B). A subset of points of ***P***^3^ contained only in ***I***^4^ are extracted as a point cloud for asymmetry analysis of ***P***^4^, as shown in Supplementary Fig. 2C.

#### Stage 5: Generate a reflected point cloud

To carry out the asymmetry analysis, a reflected point cloud ***P***^4***r***^ is first generated by taking a mirror projection of ***P***^4^ with respect to the approximated median sagittal (*xz*) plane (Supplementary Fig. 2C). Since the points close to the outer boundaries of ***P***^4^ and ***P***^4***r***^ may contain larger measurement errors than those in the center, width-restricted point clouds ***P***^5^ and ***P***^5***r***^ are extracted whose ranges in *y* direction is limited to double-sided 90% range of those of ***P***^4^ and ***P***^4***r***^. Width-restricted point cloud ***P***^5^ and its reflected version ***P***^5***r***^ are then used to find the iterative closest point (ICP)-based best fit.

#### Stage 6: Find the best fit between the two point clouds

The point-to-plane ICP algorithm^26^ is carried out to obtain an optimum position and orientation $\langle R,t\rangle$ for ***P***^5***r***^ best-fitted to ***P***^5^. As shown in Supplementary Fig. 3A, in ICP, the optimum ***R***, ***t*** is derived as a solution of a minimization problem defined in Eq. (1):

1 $$\mathop{\mathrm{minimize}\,}\limits_{\langle {\boldsymbol{R}},{\boldsymbol{t}}\rangle }\frac{1}{|\{({{\boldsymbol{p}}}_{{\boldsymbol{c}}({\boldsymbol{i}})}^{5},{{\boldsymbol{p}}}_{{\boldsymbol{i}}}^{5{\boldsymbol{r}}})\}|}\sum _{{{\boldsymbol{p}}}_{{\boldsymbol{c}}({\boldsymbol{i}})}^{5}\in {{\boldsymbol{P}}}^{5},\,{{\boldsymbol{p}}}_{{\boldsymbol{i}}}^{5{\boldsymbol{r}}}\in {{\boldsymbol{P}}}^{5{\boldsymbol{r}}}}{|{{\boldsymbol{n}}}_{{\boldsymbol{c}}({\boldsymbol{i}})}^{5}\cdot ({\boldsymbol{R}}{{\boldsymbol{p}}}_{{\boldsymbol{i}}}^{5{\boldsymbol{r}}}+{\boldsymbol{t}}-{{\boldsymbol{p}}}_{{\boldsymbol{c}}({\boldsymbol{i}})}^{5})|}^{2},$$

where $({{\boldsymbol{p}}}_{{\boldsymbol{c}}({\boldsymbol{i}})}^{5},{{\boldsymbol{p}}}_{{\boldsymbol{i}}}^{5{\boldsymbol{r}}})$ gives a valid pair of the closest points on ***P***^5^ and ***P***^5***r***^, ***R*** is a 3 × 3 rotation matrix, and ***t*** represents a 3D translation vector for transforming reflected point cloud ***P***^5***r***^. $|\{({{\boldsymbol{p}}}_{{\boldsymbol{c}}({\boldsymbol{i}})}^{5},\,{{\boldsymbol{p}}}_{{\boldsymbol{i}}}^{5{\boldsymbol{r}}})\}|$ denotes the number of valid pairs of the closest points. A normal vector ${{\boldsymbol{n}}}_{{\boldsymbol{i}}}^{5}$ and a radius of curvature ${{\boldsymbol{\rho }}}_{{\boldsymbol{i}}}^{5}$at every point ${{\boldsymbol{p}}}_{{\boldsymbol{i}}}^{5}$ in ***P***^5^, and ${{\boldsymbol{n}}}_{{\boldsymbol{i}}}^{5{\boldsymbol{r}}}$ and ${{\boldsymbol{\rho }}}_{{\boldsymbol{i}}}^{5{\boldsymbol{r}}}$at ${{\boldsymbol{p}}}_{{\boldsymbol{i}}}^{5{\boldsymbol{r}}}$ in ***P***^5***r***^ are precomputed by principal component analysis using a set of neighboring points ${\boldsymbol{N}}({{\boldsymbol{p}}}_{{\boldsymbol{i}}}^{5},{{\boldsymbol{r}}}^{5})$ and ${\boldsymbol{N}}({{\boldsymbol{p}}}_{{\boldsymbol{i}}}^{5{\boldsymbol{r}}},{{\boldsymbol{r}}}^{5})$ respectively, where ***N***(***p***, ***r***) is a set of points included in a small sphere centered at ***p*** with a radius ***r***. ***r***^5^ = 25 *mm* has been shown to give adequate results.

To avoid to taking an improper (noncorresponding) point pair $({{\boldsymbol{p}}}_{{\boldsymbol{c}}({\boldsymbol{i}})}^{5},{{\boldsymbol{p}}}_{{\boldsymbol{i}}}^{5{\boldsymbol{r}}})$ in evaluating Eq. (1), if the angle between the normal vectors at the closest point pairs $({{\boldsymbol{n}}}_{{\boldsymbol{c}}({\boldsymbol{i}})}^{5},{{\boldsymbol{n}}}_{{\boldsymbol{i}}}^{5{\boldsymbol{r}}})$ exceeds the threshold (50°), such a point pair is treated as invalid and is ignored in minimizing Eq. (1).

Unfortunately, $\langle R,t\rangle$ derived from Eq. (1) may change slightly depending on the initial orientation of ***P***^5***r***^ relative to ***P***^5^, and this change may induce a non-negligible error in the asymmetry analysis. To avoid this, we perform small rotational perturbations from −5° to 5° at intervals of 1.25° about the *x* and *y* axes to the initial orientation of ${{\boldsymbol{P}}}_{{\boldsymbol{r}}}^{5}$ and then conduct ICP on Eq. (1) several times. In each optimization process from the initial orientation, we iterate the process 50 times. Finally, the best fitted $\langle R,t\rangle$, which gives the smallest value of Eq. (1), is adopted as the optimum rotation and translation $\langle {R}^{\ast },{t}^{\ast }\rangle$ for ***P***^5***r***^, as shown in Supplementary Fig. 3B. By applying $\langle {R}^{\ast },{t}^{\ast }\rangle$ to the original reflected point cloud ***P***^4***r***^, the optimal reflected point cloud ***P***^6***r***^—which is best fitted to the original point cloud ***P***^4^—is finally generated (Supplementary Fig. 3C).

#### Stage 7: Roughly extract deviations for colormap rendering

The difference in the position of ***P***^6***r***^ relative to ***P***^4^ is roughly evaluated, and the difference distribution is rendered as a color map as Supplementary Fig. 4. In the evaluation, a triangle mesh surface ***M***^6***r***^ is generated from point cloud ***P***^6***r***^. Then, distance ${{\boldsymbol{d}}}_{{\boldsymbol{i}}}^{4}$ is evaluated from a point ${{\boldsymbol{p}}}_{{\boldsymbol{i}}}^{4}$ in ***P***^4^ to the point on ***M***^6***r***^ closest to ${{\boldsymbol{p}}}_{{\boldsymbol{i}}}^{4}$ along the normal vector ${{\boldsymbol{n}}}_{{\boldsymbol{i}}}^{4}$ at ${{\boldsymbol{p}}}_{{\boldsymbol{i}}}^{4}$. If ${{\boldsymbol{d}}}_{{\boldsymbol{i}}}^{4}$ exceeds the threshold (100 mm), it is ignored in the evaluation. Finally, the distribution of the distances $\{{{\boldsymbol{d}}}_{{\boldsymbol{i}}}^{4}\}$ is rendered as a colormap on ***P***^4^ representing how much the geometry of the surface of the subject’s back deviates from its ideally symmetric shape, and where the deviations are most prominent.

#### Stage 8: Precisely extract deviations and estimate the asymmetry index

Finally, the asymmetry index is evaluated, which precisely indicates the extent to which the geometry of the surface of the patient’s back deviates from perfect symmety. For this purpose, the centroid and principal axes of the union of point clouds ***P***^6***r***^ and ***P***^4^ are calculated (Supplementary Fig. 5A), and the points clouds ***P***^4^ and ***P***^6***r***^ are transformed to ***P***^7^ and ***P***^7***r***^ such that the principal axes are aligned to *x, y*, and *z* axes where the *xy* plane is aligned to the frontal plane and the *x* axis is oriented to the craniocaudal axis. Then, as shown in Supplementary Fig. 5B, two point clouds ***P***^7^ and ***P***^7***r***^ are regularly resampled at two-dimensional rectangular grid points, each of which (***l***, ***m***) is located on the *xy* plane and whose grid spacings are ∆*x* and ∆*y* in the *x* and *y* directions of the new axes. As a result, two resampled point clouds,

$$\begin{array}{c}{{\boldsymbol{P}}}^{{\bf{8}}}=\{{{\boldsymbol{p}}}_{{\boldsymbol{l}},{\boldsymbol{m}}}^{{\bf{8}}}=({{\boldsymbol{p}}}_{{\boldsymbol{x}},\,{\boldsymbol{l}},{\boldsymbol{m}}}^{{\bf{8}}},\,{{\boldsymbol{p}}}_{{\boldsymbol{y}},{\boldsymbol{l}},{\boldsymbol{m}}}^{{\bf{8}}},{{\boldsymbol{p}}}_{{\boldsymbol{z}},{\boldsymbol{l}},{\boldsymbol{m}}}^{{\bf{8}}})|{\boldsymbol{l}}=\frac{{\boldsymbol{x}}}{{\rm{\Delta }}{\boldsymbol{x}}},{\boldsymbol{m}}=\frac{{\boldsymbol{y}}}{{\rm{\Delta }}{\boldsymbol{y}}}\}\,\,{\rm{and}}\\ {{\boldsymbol{P}}}^{{\bf{8}}{\boldsymbol{r}}}=\{{{\boldsymbol{p}}}_{{\boldsymbol{l}},{\boldsymbol{m}}}^{{\bf{8}}{\boldsymbol{r}}}=({{\boldsymbol{p}}}_{{\boldsymbol{x}},{\boldsymbol{l}},{\boldsymbol{m}}}^{{\bf{8}}{\boldsymbol{r}}},{{\boldsymbol{p}}}_{{\boldsymbol{y}},{\boldsymbol{l}},{\boldsymbol{m}}}^{{\bf{8}}{\boldsymbol{r}}},{{\boldsymbol{p}}}_{{\boldsymbol{z}},{\boldsymbol{l}},{\boldsymbol{m}}}^{{\bf{8}}{\boldsymbol{r}}})|{\boldsymbol{l}}=\frac{{\boldsymbol{x}}}{{\rm{\Delta }}{\boldsymbol{x}}},{\boldsymbol{m}}=\frac{{\boldsymbol{y}}}{{\rm{\Delta }}{\boldsymbol{y}}}\},\end{array}$$

are generated from ***P***^7^ and ***P***^7***r***^, respectively. We adopted 3 mm as ∆*x* and ∆*y* based on the space sampling resolution of our depth sensor. Usually, resampled points ${{\boldsymbol{p}}}_{{\boldsymbol{l}},{\boldsymbol{m}}}^{8}$ and ${{\boldsymbol{p}}}_{{\boldsymbol{l}},{\boldsymbol{m}}}^{8{\boldsymbol{r}}}$ do not exactly locate on points in ***P***^7^ and ***P***^7***r***^. Therefore, as shown in Supplementary Fig. 5C, we first find a point ${{\boldsymbol{p}}}_{{\boldsymbol{j}}}^{7}$in ***P***^7^ whose projection on the *xy* plane is closest to $({{\boldsymbol{p}}}_{{\boldsymbol{x}},{\boldsymbol{l}},{\boldsymbol{m}}}^{8},{{\boldsymbol{p}}}_{{\boldsymbol{y}},{\boldsymbol{l}},{\boldsymbol{m}}}^{8},0)$, and then fit a small spherical surface ***S***, which passes through ${{\boldsymbol{p}}}_{{\boldsymbol{j}}}^{7}$ and has a normal vector ${{\boldsymbol{n}}}_{{\boldsymbol{j}}}^{7}$ and radius of curvature ${{\boldsymbol{\rho }}}_{{\boldsymbol{j}}}^{7}$ at ${{\boldsymbol{p}}}_{{\boldsymbol{j}}}^{7}$, which was computed in Step 6. Finally, we find a resampled point ${{\boldsymbol{p}}}_{{\boldsymbol{l}},{\boldsymbol{m}}}^{8}$ intersecting a vertical line passing $({{\boldsymbol{p}}}_{{\boldsymbol{x}},{\boldsymbol{l}},{\boldsymbol{m}}}^{8},{{\boldsymbol{p}}}_{{\boldsymbol{y}},{\boldsymbol{l}},{\boldsymbol{m}}}^{8},0)$ and surface ***S*** to build ***P***^8^. The other resampled point ${{\boldsymbol{p}}}_{{\boldsymbol{l}},{\boldsymbol{m}}}^{8{\boldsymbol{r}}}$ in ***P***^8***r***^ is calculated similarly.

An example of ***P***^7^ and ***P***^8^ is shown in Supplementary Fig. 6. If both resampled points ${{\boldsymbol{p}}}_{{\boldsymbol{l}},{\boldsymbol{m}}}^{8}$ and ${{\boldsymbol{p}}}_{{\boldsymbol{l}},{\boldsymbol{m}}}^{8{\boldsymbol{r}}}$ lie inside the effective range of ***P***^8^ and ***P***^8***r***^ at a grid point (***l***, ***m***), and these points can be resampled correctly, then grid point (***l***, ***m***) is treated as effective, and is included in effective grid point set ***G***_***E***_.

As shown in Supplementary Fig. 7, a new normal vector ${{\boldsymbol{n}}}_{{\boldsymbol{l}},{\boldsymbol{m}}}^{8}$ at ${{\boldsymbol{p}}}_{{\boldsymbol{l}},{\boldsymbol{m}}}^{8}$ is estimated using the principal component analysis of the neighboring point set ${\boldsymbol{N}}({{\boldsymbol{p}}}_{{\boldsymbol{l}},{\boldsymbol{m}}}^{8},{{\boldsymbol{r}}}^{8})$. Plane ***S***′, which passes through ${{\boldsymbol{p}}}_{{\boldsymbol{l}},{\boldsymbol{m}}}^{8}$ and has a normal ${{\boldsymbol{n}}}_{{\boldsymbol{l}},{\boldsymbol{m}}}^{8}$, is generated, and distance ***d***_***l***, ***m***_ between plane ***S***′ and ${{\boldsymbol{p}}}_{{\boldsymbol{l}},{\boldsymbol{m}}}^{8{\boldsymbol{r}}}$ is evaluated as a *deviation* at this resampled point. ***r***^8^ = 25 mm gives adequate results. However, even if grid point (***l***, ***m***) is included in effective grid point set ***G***_***E***_, if (***l***, ***m***) lies close to the boundary of ***P***^8^, it is possible that resampled points ${{\boldsymbol{p}}}_{{\boldsymbol{l}},{\boldsymbol{m}}}^{8{\boldsymbol{r}}}$ or ${{\boldsymbol{p}}}_{{\boldsymbol{l}},{\boldsymbol{m}}}^{8}$ still suffer from relatively large measurement error due to the high incident angle between the optical axis of the depth sensor and the surface normal. Therefore, we must evaluate deviation ***d***_***l***, ***m***_ only at a valid grid point (***l***, ***m***), which is included in ***G***_***E***_ but does not lie close to the boundary of ***P***^8^. To do so, as shown in Supplementary Fig. 8, we adopt grid point (***l***, ***m***)as valid only if it lies in a valid range located around the median sagittal plane such as ***M***_***min***_(***l***) + 0.5(1 − ***α***)***W***(***l***) ≤ ***m*** ≤ ***M***_***max***_(***l***) − 0.5(1 − ***α***)***W***(***l***), where ***M***_***min***_(***l***) and ***M***_***max***_(***l***) are minimum and maximum values of the *y* coordinate of the grid points at ***l*** included in ***G***_***E***_, and ***W***(***l***) = ***M***_***max***_(***l***) − ***M***_***min***_(***l***). The parameter ***α ***∈ [0, 1] controls to what extent we adopt the grid points in ***G***_***E***_ as valid ones for the final asymmetry analysis. Based on this criterion, if a grid point (***l***, ***m***) is classified as valid, then the point is included in valid grid point set ***G***_***v***_.

Finally, the distribution of the sampled deviation between the point clouds of the surface of the patient’s back ***P***^8^ and its best-fitted and reflected point cloud ***P***^8***r***^ at valid grid points is determined as ${\boldsymbol{D}}=$ $\{{{\boldsymbol{d}}}_{{\boldsymbol{l}},{\boldsymbol{m}}}|{\boldsymbol{l}}=\lfloor \frac{{\boldsymbol{x}}}{{\rm{\Delta }}{\boldsymbol{x}}}\rfloor ,{\boldsymbol{m}}=\lfloor \frac{{\boldsymbol{y}}}{{\rm{\Delta }}{\boldsymbol{y}}}\rfloor ,({\boldsymbol{l}},{\boldsymbol{m}})\in {{\boldsymbol{G}}}_{{\boldsymbol{v}}}\}$. Based on distribution ***D***, the *asymmetry index* ***I***_***asym***_ is evaluated as an averaged deviation over the grid points included in valid grid point set ***G***_***v***_ using Eq. (2), which precisely represents the degree of asymmetry quantitatively:

2 $${{\boldsymbol{I}}}_{{\boldsymbol{asym}}}=\frac{1}{|{{\boldsymbol{G}}}_{{\boldsymbol{v}}}|}\,\sum _{({\boldsymbol{i}},{\boldsymbol{j}})\in {{\boldsymbol{G}}}_{{\boldsymbol{v}}}}\,{{\boldsymbol{d}}}_{{\boldsymbol{i}},{\boldsymbol{j}}},$$

where |***G***_***v***_| denotes the number of valid grid points. If the shape of the back surface at ***G***_***v***_ is exactly symmetric with respect to the median sagittal plane, index ***I***_***asym***_ is zero; the more the back’s shape deviates from perfect symmetry, the greater ***I***_***asym***_ becomes.

### Capturing Posture Images

For this fully automated algorithm for 3D asymmetry detection, the subject is asked to bend forward, a pose in which protrusions on the back are most easily observed and which is extensively used for the traditional scoliosis examination known as the Adams bending test^15,16^. To prevent cases in which the patient’s posture diverges significantly from the recommended pose, such as cases in which the subject’s back is not visible in the photographed data, the system implements the following countermeasures: (1) The positions in which subjects should place their feet are marked. In addition, a box that indicates the recommended positioning is displayed on the screen during photography. The subject can be positioned based on this frame and visually guided to approach the recommended position in the x (left–right) and y (craniocaudal) directions, and attain the proper z rotation angle. (2) The angular difference between the approximate plane of the 3D point cloud in the detection region of the photographed subject, and the direction of photography of the 3D sensor is calculated during photography. This value can be checked to assess whether it is within the threshold value; via analysis of the 3D data, the rotation angles along the x direction (<±7.5°), and the angular rotation along the y direction (<±15°) can be made to approach the recommended state.

### Human Subjects

Informed consent was obtained from all the parents/legal guardians of paediatric patients and all work was performed in accordance with the guidelines of the institutional internal review board of the participating institution. The experimental protocols were approved by the institutional review board of Hokkaido University Hospital, and the registered number is 016–0236. The human subjects had been referred to our hospital on suspicion of IS and were asked to bend forward, as for the Adam’s forward bending test^15,16^, followed by scanning with the sensor. The inclusion criteria were as follows: (i) aged 7 to 18 years old; (ii) referred for confirmed diagnosis based on x-ray; (iii) willing and able to provide written informed consent. We explained the procedure, indications, and preparation, and participants indicated their understanding and signed the corresponding consent forms. We received the subjects’ medical records to comparing the accuracy of the developed technique with the current standard of care. The medical records included age, sex, body mass index (BMI), and Cobb angle measured from radiographs. Seventy-six 3D point clouds and Cobb angles were obtained for this study. The average age was 13.8 years (range, 7 to 18 years). The mean BMI and Cobb angle was 18.6 (range, 13.4 to 27.0) and 21.2° (range, 0° to 64°), respectively. When subjects with a Cobb angle of 0° were excluded, the mean Cobb angle of single thoracic curve (n = 33), double thoracic and thoracolumbar/lumbar curve (n = 19), and single thoracolumbar/lumbar (n = 17) was 22.7° (range, 4° to 64°), 29.1° (range, 18° to 43°) and 25.2° (range, 12° to 41°), and 18.2° (range, 8° to 35°), respectively. Our internal studies of intra- and inter-rater reliability have shown excellent kappa statistics^27^ for the Cobb angle measurements (0.95–0.99).

### Repeatability Analysis and the Effects of Trunk Rotation

To assess the independent repeatability of and effects of trunk rotation on this system isolated from human subjects, phantom models were obtained from plaster wrap castings made when IS patients were fitted for hard braces to treat single thoracic curves, double thoracic and thoracolumbar/lumbar curves, and single thoracolumbar/lumbar curves, respectively. The phantom models were scanned 10 times from the front, and at 5° clockwise and counterclockwise rotations.

### Intra-observer repeatability

To assess intra-observer repeatability, thirty subjects who agreed to the repeated test were scanned in the same position. The test-retest evaluation was performed in two acquisitions for the same subject, with repositioning between the acquisitions.

### Effects of smoothing operations on the point clouds

We have assessed the precision of the method by comparing the asymmetry index between the point cloud captured from a phantom model’s surface by a high-definition non-contact 3D digitizer (Vivid 910, Konica Minolta Inc., Tokyo, Japan) and that by our consumer-grade sensor. The high-definition non-contact 3D digitizer has depth measurement range of 0.6–2.5 m; a horizontal and vertical scan range of 823 mm and 618 mm at 2.5 m depth, respectively. The measurement accuracy in the horizontal, vertical, and depth directions are ±0.38 mm, ±0.31 mm, and ±0.20 mm to the reference plane at the middle measurement mode. Around 98,000 dense points on the dummy surface were captured by the digitizer in a scan. Therefore, the accuracy of the non-contact 3D digitizer is much better than that of the 3D depth sensor, and the point clouds measured by the digitizer can be treated as reference data in the comparison. The phantom model was obtained from plaster casting when a patient was fitted for a hard brace (single thoracic curve, T5-T11,31°). We also evaluated the difference in the asymmetry index before and after our approximation, and smoothing operations for the point clouds measured both from the 3D digitizer and the consumer-grade sensor. The phantom model was scanned three times from the front.

### Statistical analysis

Pearson’s correlation coefficient analyses were used to assess relationships between the asymmetry index and the Cobb angle. A Fisher *r-to-z* transformation was used to test for the difference between two correlation coefficients. Receiver operating characteristic (ROC) analyses were used to estimate the best cut-off values for the asymmetry index to predict Cobb angles greater than 15°, 20°, or 25°. The performance of the asymmetry indices were evaluated using the area under the ROC curve (AUC). AUCs were categorized as follows: no discrimination (AUC = 0.50), acceptable discrimination (0.7 ≤ AUC < 0.8), excellent discrimination (0.8 ≤ AUC < 0.9), and outstanding discrimination (AUC ≥ 0.9)^28^. To determine the cut-off values, we used the Youden index, which is calculated as sensitivity + specificity −1 for each potential cut-off point; the optimal cut-off point is the tool score with the highest value^29^. Based on the cut-off values, the sensitivities, specificities, positive predictive values, negative predictive values, accuracy, positive likelihood ratios, and negative likelihood ratios were determined. One-way ANOVA was used to compare differences across positions. Repeatability was determined in terms of the coefficient of variation (CV), which is a measure of variability relative to the mean and is calculated by dividing the standard deviations of intrasubject results by the mean and multiplying by 100 to present the result as a percentage^30^. A CV less than 10% was regarded as very good, 10% < CV ≤ 20% as good, 20% < CV ≤ 30% as fair/moderate, and CV > 30% as poor^31^. The intraclass correlation coefficient (ICC) was calculated to evaluate intra-observer repeatability. ICC values less than 0.5 are indicative of poor reliability, values between 0.5 and 0.75 indicate moderate reliability, values between 0.75 and 0.9 indicate good reliability, and values greater than 0.90 indicate excellent reliability^32^. Data analyses, except for ICC, were performed using JMP statistical software for Windows (version 12; SAS, Inc., Cary, NC, USA). The ICC was analysed using SPSS software for Windows (version 23; IBM, Inc., Chicago, IL, USA). *P* < 0.05 was considered statistically significant.

## Results

### Evaluation of back surface asymmetricity

**Figure 2:**
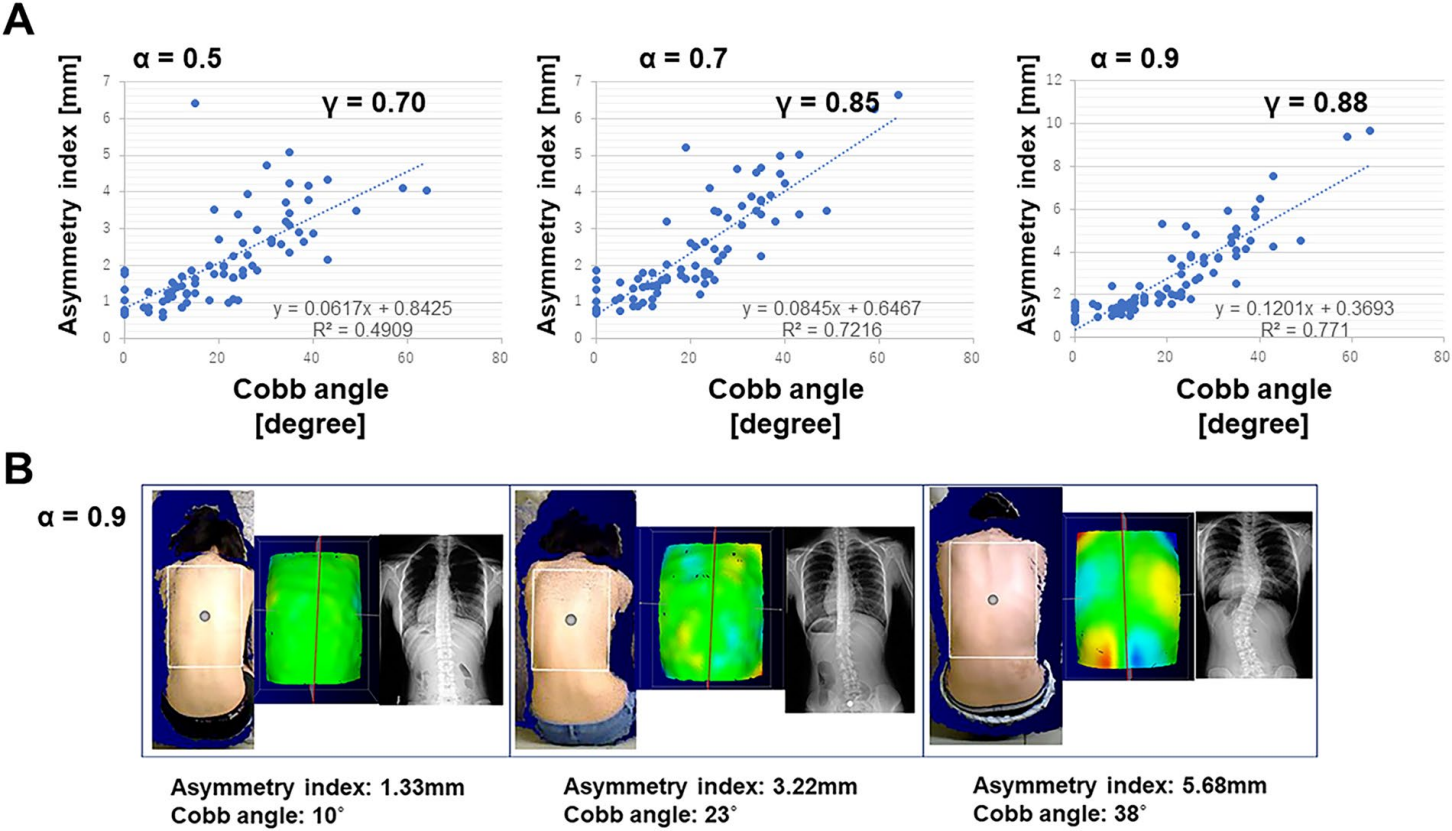
Correlation between the asymmetry index and Cobb angle. (**A**) = 1.0 if defined as the distance from the narrowest part of the waist to the centerline. (**B**) Comparison to X-ray photos. Deviations in the image correspond with curvatures. The system evaluates the degree to which a patient’s back deviates from the ideal symmetry for a human back. The larger the deviation, the deeper the color.

To evaluate the ability of our algorithm to distinguish asymmetric back surfaces and to reveal scoliosis lesions throughout the spine, we obtained 3D point cloud data retrieved from a 3D depth sensor from 76 subjects (18 males and 58 females) who satisfied the study enrollment criteria. The time from scanning to obtaining an analysis result was 1.5 ± 0.4 s (range, 0.4 to 2.8 s). For qualitative evaluation, the asymmetry index was compared with the Cobb angle. The coefficients of correlation were evaluated at α = 0.5, 0.7, and 0.9 from the center shaft, respectively. When α = 0.9 from the central shaft was adopted, the coefficient of correlation was 0.88 (Fig. 2). Based on these results, we set α = 0.9 for further analyses. There was no significant difference of the correlation coefficient between male (*r* = 0.92) and female (*r* = 0.86) (*p* = 0.26). In addition, the asymmetry index was compared with the BMI. The coefficient of correlation was 0.10 (*p* = 0.40). When subjects with a Cobb angle of 0° were excluded, there was no significant difference of the correlation coefficient between single curve (n = 50, *r* = 0.89) and double curve (n = 19, *r* = 0.85) (*p* = 0.49). However, there was significant difference of the correlation coefficient between single thoracic curve (n = 33, *r* = 0.92) and single thoracolumbar/lumbar curve (n = 17, *r* = 0.68) (*p* = 0.02).

### Scoliosis Predictions

We evaluated the ability of our system to predict IS (Table 1). The ROC curves were plotted using the asymmetry index and showed that the cut-off value of the asymmetry index was 3.28 when a Cobb angle of greater than 25° was selected for patients requiring brace treatment. To predict an IS angle greater than 25°, the AUC was 0.96, sensitivity was 0.97, specificity was 0.88, positive predictive value was 0.82, negative predictive value was 0.98, accuracy was 0.91, positive likelihood ratio was 8.00, and negative likelihood ratio was 0.04. The AUC was regarded as having outstanding discrimination. Results based on Cobb angles of 15° and 20° are also shown in Table 1. Furthermore, results of an ROC analysis with breakdown of the curve type are shown in the Table 1.

**Table 1:** ROC analysis with Cobb angle.

Curve Type	Cobb angle	Cut-off value	AUC (95% CI)	Sensitivity	Specificity	PPV	NPV	Accuracy	PLR	NLR
Total subjects	15°	2.67	0.92 (0.85, 0.96)	0.79	0.92	0.93	0.76	0.84	9.99	0.23
	20°	2.79	0.94 (0.88, 0.97)	0.89	0.91	0.91	0.89	0.90	10.20	0.10
	25°	3.28	0.96 (0.91, 0.99)	0.97	0.88	0.82	0.98	0.91	8.00	0.04
Single thoracic curve	15°	1.63	0.99 (0.90, 1.00)	1.00	0.93	0.95	1.00	0.97	14.00	0.00
	20°	1.82	0.96 (0.78, 0.99)	1.00	0.88	0.89	1.00	0.94	8.50	0.00
	25°	1.82	0.93 (0.76, 0.98)	1.00	0.83	0.83	1.00	0.91	6.00	0.00
Double thoracic and thoracolumbar/lumbar curve	15°	—	—	—	—	—	—	—	—	—
	20°	2.45	0.98 (0.73, 1.00)	0.94	1.00	1.00	0.75	0.95	∞	0.06
	25°	3.75	0.96 (0.78, 1.00)	0.83	1.00	1.00	0.78	0.90	∞	0.17
Single thoracolumbar/lumbar curve	15°	1.86	0.79 (0.43, 0.95)	0.90	0.71	0.82	0.83	0.82	3.15	0.14
	20°	1.97	0.82 (0.52, 0.95)	0.88	0.78	0.78	0.88	0.82	3.94	0.16
	25°	2.50	0.97 (0.62, 1.00)	0.67	1.00	1.00	0.93	0.94	∞	0.33

AUC = area under the curve, PPV = positive predictive value, NPV = negative predictive value, PLR = positive likelihood ratio, NLR = negative likelihood ratio, — = not applicable, ∞ = infinity.

### Repeatability Analysis and Effects of Trunk Rotation

The asymmetry index and CV for each phantom model after 10 trials is shown in Table 2. There was a significant difference between the forward view and 5° rotations in each model (*p* < 0.05); however, these difference were small and due to the low standard deviations. The CV was 1–4%, which is regarded as very good repeatability.

**Table 2:** Radiographic Parameters.

	Front	Clockwise 5°	Counterclockwise 5°	Overall *P* value	Tukey *P* value		
					Front vs Clockwise	Clockwise vs Counterclockwise	Front vs Counterclockwise
Single thoracic curve (T7-L2, 40°)							
Asymmetry index (mm)	2.13 ± 0.04	2.16 ± 0.02	2.19 ± 0.03	0.005	0.004	0.089	0.389
Coefficient of variation (%)	2	1	1				
Double thoracic and thoracolumbar/lumbar curve (T6-L1-L4, 38°–25°)							
Asymmetry index (mm)	2.10 ± 0.09	2.14 ± 0.07	2.21 ± 0.05	0.031	0.448	0.282	0.024
Coefficient of variation (%)	4	3	2				
Single thoracolumbar/lumbar curve (T11-L4, 38°)							
Asymmetry index (mm)	2.89 ± 0.02	2.95 ± 0.03	2.98 ± 0.04	<0.001	0.003	0.075	<0.001
Coefficient of variation (%)	1	1	1				

The values of asymmetry index are given as the average and the standard deviation.

### Intra-observer repeatability

The ICC obtained for intra-observer repeatability for human subjects was 0.995 (95% confidence interval: 0.989–0.997), which is regarded as excellent intra-observer repeatability.

### Effects of smoothing operations on the point clouds

While there is no significant difference in the asymmetry index before and after the operations of the point cloud obtained from the a high-definition non-contact 3D digitizer, a significant difference is observed in the indexes when using the consumer-grade sensor. In addition, a significant difference was observed in the asymmetry index between the high-definition non-contact 3D digitizer and the consumer-grade sensor (Table 3).

**Table 3:** Asymmetry index obtained from high-definition depth sensor before and after filtering of the point cloud.

	High-definition depth sensor (Vivid)			Consumer-grade depth sensor (Xtion)			Vivid (Before filtering) vs Xtion (After filtering)
	Before filtering	After filtering	*P* value	Before filtering	After filtering	*P* value	*P* value
Asymmetry index (mm)	1.72 ± 0.01	1.73 ± 0.03	0.74	2.63 ± 0.01	2.15 ± 0.04	<0.01	<0.01

The values of asymmetry index are given as the average and the standard deviation.

## Discussion

Recently, the feasibility and potential of using 3D US imaging methods has been demonstrated for measuring coronal deformities in patients with AIS^17,18^. Although manual angle measurements appear very repeatable, they remains subjective and relatively time-consuming, as the imaging probe must be manually used on the body^17,18^. Conversely, surface topography asymmetry maps have been suggested as a noncontact technique for monitoring IS curve progression^2,33,34^. Although points representing the arms, hips, head, and neck must be manually deleted and the comparison between the obtained asymmetry map and the deformities measured via x-ray require further investigation, this study shows promise in finding the best plane of symmetry, which is defined as the plane that produces the minimum distance between the points of the original torso and corresponding points of the reflected torso^33,34^. For each point on the original torso, its corresponding point is the closest point on the reflected torso; however, the optimized reflected torso relies on the initial settings of the original torso^33,34^.

In our algorithm, several default positions of the reflected torso are first generated on the approximated midsagittal plane. Then, the square distance of corresponding point clouds between the original and reflected torsos is repeatedly minimized using ICP methods^26^, and the result with the minimum square distance is selected, enabling stable results independent of the difference in the default position. Therefore, this algorithm does not require strict pose matching. This device was not developed to eliminate the impact of the patient’s posture on the results through strict positioning of the patient; instead, the patient was allowed to approximate the recommended pose, which may lead to some variation in posture and position. Therefore, the 3D curved surface data of the patient’s back is commonly obtained with the torso in a rotated condition.

However, in the proposed algorithm, the original curved surface data and the reflected curved surface data are calculated, and the 3D transformation that yields the closest match between this reflected curved surface and the original curved surface is calculated. In other words, the optimal 3D translation and rotation angles are calculated with respect to the reflected curved surface that minimizes the sum-of-the-squares of the distances between corresponding points on the two curved surfaces. The algorithm applies a coordinate transformation to the position and posture equal to this optimal translation and a rotational transformation that produces the best fit between the original and the reflected curved surface data. Based on the reflected curved surface data after coordinate transformation (i.e., the best-fitting curved surface), 3D asymmetry is calculated using the difference between the original and reflected curved surface data.

Therefore, even if the original curved surface data measured from the patient’s back is tilted to the left or right, applying the best fit processing described above also rotates the reflected curved surface data, and maintains the best fit in reference to the original curved surface data. As a result, the position and posture of the reflected curved surface data, which constitute the basis for evaluation of the asymmetry, depend mostly on the curved surface shape of the original 3D curved surface data, and are uniquely determined. Therefore, the results are almost unaffected by the patient’s position or orientation.

As described above, the principal reliability of the data is assured by the algorithm’s intrinsic reliability and through various countermeasures. However, point cloud alignments depend on the quality of the 3D depth sensor that effects reproducible measurement. Therefore, the calculation of the asymmetry metric uses 3D data from 90% of the center shaft and does not use data from the edges of the surface of the back.

Surface metrics have very little correlation to Cobb angle measurements^21^; indices measured on different planes do not correlate^21^. Different indices exhibit divergent characteristics in observer-induced errors, accuracy, sensitivity, and specificity^21^. Complicated positioning of the patient and ambiguous anatomical landmarks are major sources of error^21^. Because trunk asymmetry does not significantly correlate with scoliosis^35,36^, the Adams forward bending test is carried out to distinguish between faulty posture and actual idiopathic scoliosis^11^. Currently, the scoliometer with the Adams forward bending test is the best tool available for scoliosis screening. Scanning the body surface in the standing position has a significant limitation in the detection of scoliosis because the hump is not prominent under the posture. Our newly developed system can automatically calculate an asymmetry index, as well as a deviation contour map, and shows discriminative ability in determining whether an examinee requires an additional x-ray to confirm diagnosis. The intraclass correlation coefficient obtained for intra-observer repeatability for human subjects was 0.995, indicating that there was no significant difference in asymmetry index between the acquisitions. In addition, the system’s specificity and sensitivity did not vary according to the curve types and severity, except for cases of a mild single thoracolumbar/lumbar curve. The correlation coefficient between the asymmetry index and the Cobb angle (*r* = 0.68) was almost identical to that of a previous study which reported a correlation coefficient of 0.677^37^ between the Cobb angle and scoliometer value. Hence, the present system is limited in its ability to detect the single thoracolumbar/lumbar curves as a scoliometer would.

In this study, there is no significant difference in the asymmetry index before and after the operations of the point cloud obtained from the a high-definition non-contact 3D digitizer. This indicates that the amount of accidental error is small and negligible in the measurement by the 3D digitizer, and our smoothing operation does not affect the evaluation of the asymmetry index in cases where the accidental error in measurement is very small.

On the other hand, a significant difference in the index is observed before and after them when using the consumer-grade sensor. It was also observed that the index value after the smoothing operations of the point cloud in the consumer-grade sensor was closer to that of the 3D digitizer than the value before smoothing. This suggests that a non-negligible amount of the accidental error in the measurement was imposed on the point clouds by the consumer-grade sensor, but that the smoothing operation of the point clouds is effective for reducing the influence of these accidental errors on the asymmetry index evaluation.

In addition, while the coefficient of correlation still maintains a strong correlation level (r = 0.88) between the asymmetry index and Cobb angle when using the consumer-grade sensor, the significant difference in the asymmetry index value was observed between the high-definition non-contact 3D digitizer and the consumer-grade sensor. The difference is considered to be caused by an inescapable systematic error inherent to this consumer-grade sensor. This suggests that the cut-off value of the asymmetry index for screening must be re-adjusted if the type of the depth sensor and its measuring accuracy performance have been changed in the system. The elimination of need for the re-adjustment of the cut-off value remains as a challenge for the future.

In summary, we have developed an inexpensive and rapid 3D symmetry-recognition system for the surface of the human back that can detect early IS without the help of a specialist doctor. The system scans multiple points on the back, evaluating the back’s symmetry in a few seconds. The system now requires clinical pilot validation study so that it can be used routinely, preventing unnecessary x-rays for normal anatomies and mild case of IS, and identifying moderate IS might that otherwise be missed in clinics or physical examination at schools. This further feedback and evaluation will provide technical validation of the algorithm and workflow.

## Electronic supplementary material

Supplementary information

## Data Availability

The data that support the findings of this study are available from the corresponding author on request.
