# Supplementary material for: Automated noninvasive detection of idiopathic scoliosis in children and adolescents: A principle validation study
Source: Sci Rep. 2018 Dec 7;8:17714. doi: 10.1038/s41598-018-36360-w (PMC6286333; doi:10.1038/s41598-018-36360-w)
Supplement: Supplementary file 1 — Supplementary information [file 41598_2018_36360_MOESM1_ESM.doc]

**Automated noninvasive detection of idiopathic scoliosis in children and adolescents: A principle validation study**

Hideki Sudo^1,2*^, Terufumi Kokabu^2*^, Yuichiro Abe^3^, Akira Iwata^2^, Katsuhisa Yamada^2^, Yoichi M. Ito^4^, Norimasa Iwasaki^2^, and Satoshi Kanai^5^

^1^Department of Advanced Medicine for Spine and Spinal Cord Disorders, Faculty of Medicine and Graduate of Medicine, Hokkaido University, N15W7, Sapporo, Hokkaido 060-8638, Japan

^2^Department of Orthopaedic Surgery, Hokkaido University Hospital, N14W5, Sapporo, Hokkaido 060-8648, Japan

^3^Department of Orthopaedic Surgery, Eniwa Hospital, Koganechuo 2-1-1, Eniwa, Hokkaido 061-1449, Japan

^4^Department of Biostatistics, Hokkaido University Graduate School of Medicine, Sapporo, Hokkaido, Japan

^5^Division of Systems Science and Informatics, Hokkaido University Graduate School of Information Science and Technology, N14W9, Sapporo, Hokkaido 060-0814, Japan

*These authors contributed equally to this work.

Correspondence: Hideki Sudo, MD, PhD

Department of Advanced Medicine for Spine and Spinal Cord Disorders, Faculty of Medicine and Graduate of Medicine, Hokkaido University

N15W7, Sapporo, Hokkaido 060-8638, Japan

Tel: 81-11-706-5934 Fax: 81-11-706-6054

Email: [hidekisudo@yahoo.co.jp](mailto:hidekisudo@yahoo.co.jp)

**Supplementary figures**

**
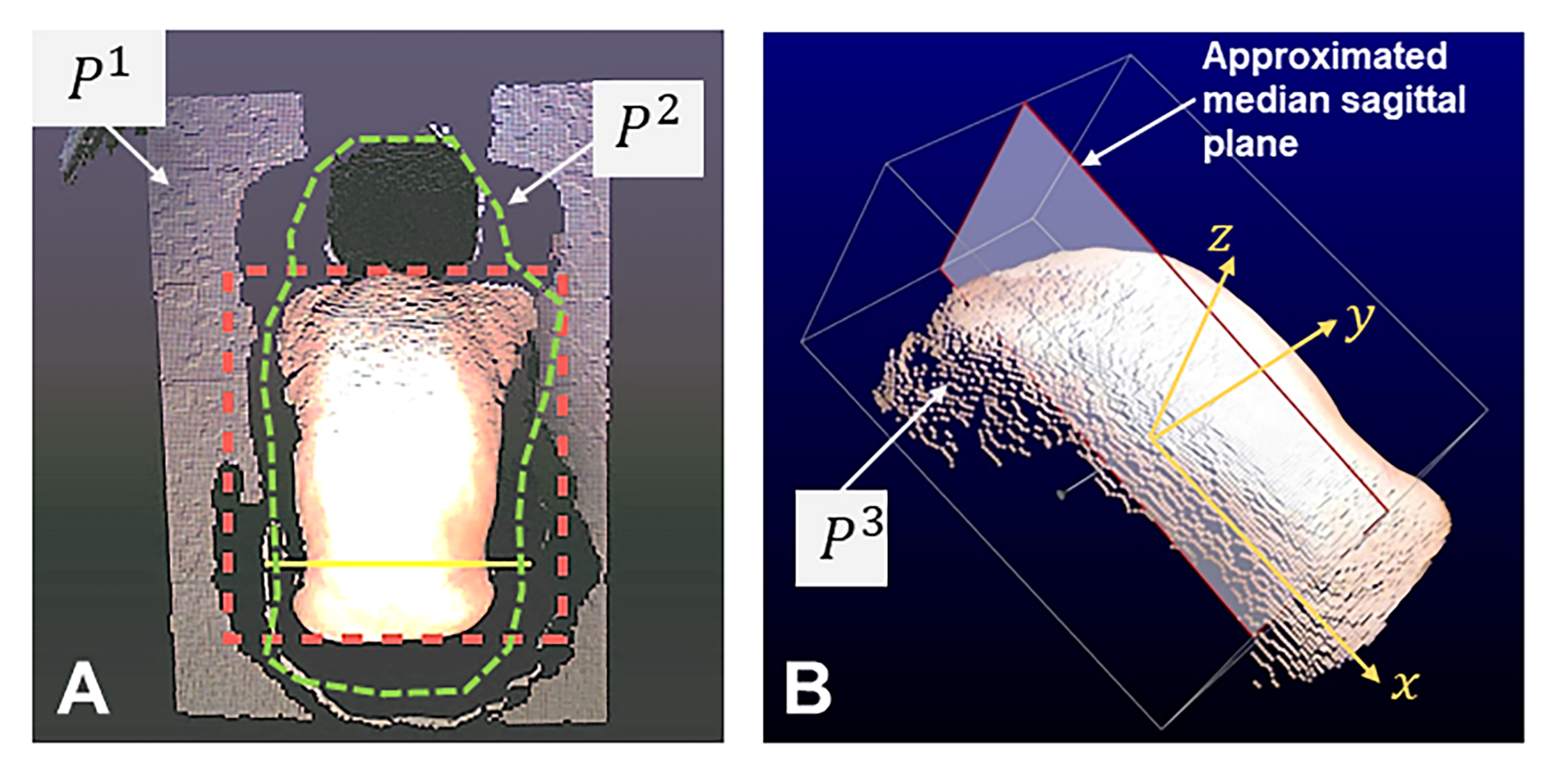
**

**Supplementary figure 1.** Capturing point clouds of a patient’s back, segmentation of the point cloud, and estimating an approximated median sagittal plane. **(A)** Original point cloud
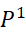
, and the point cloud including back, breech, neck and occipital surfaces
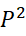
. **(B)** The pose-normalized point cloud
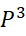
, and approximated median sagittal plane.


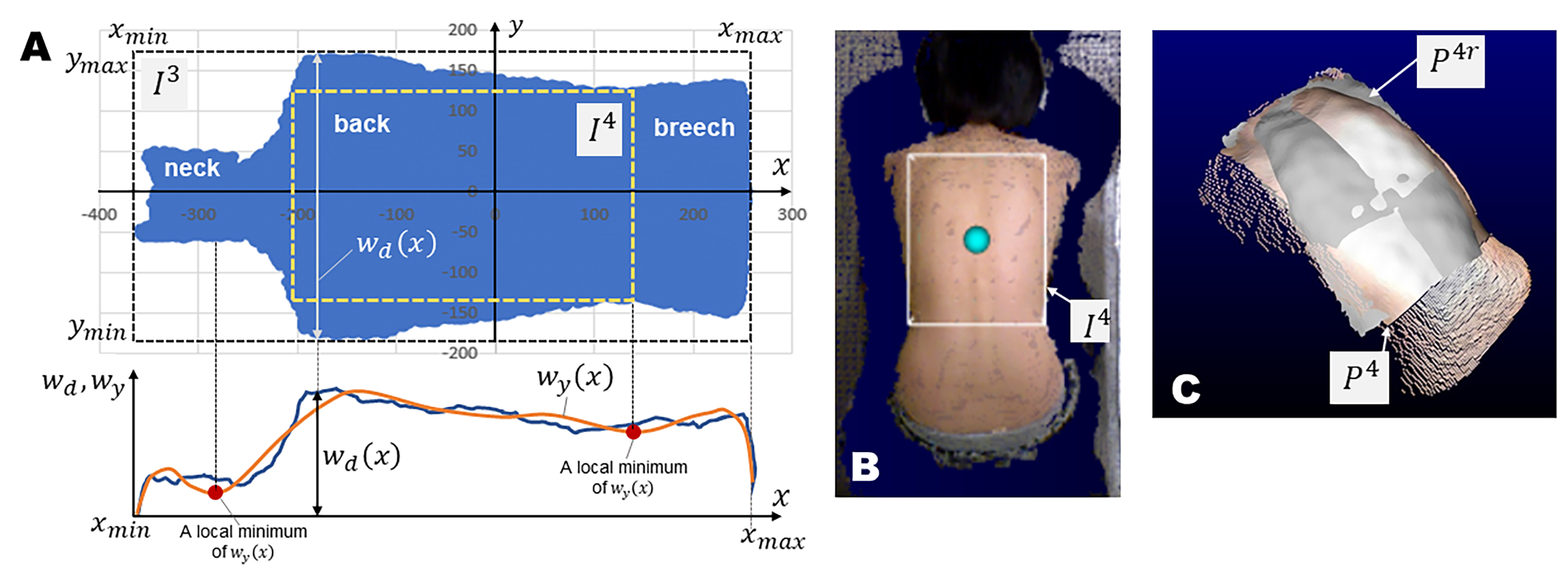


**Supplementary figure 2.** Estimating the boundaries of the surface of the back. **(A)** Estimating the boundaries of the surface of the back using binary image
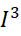
, discrete width function
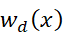
, a high-order polynomial of
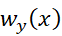
, local minima, and the identified rectangular region for analysis
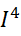
. **(B)** An example of the automatically identified rectangular region for analysis
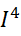
. **(C)** The point cloud for asymmetry analysis
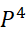
, and its reflected point cloud
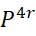
 about the approximated median sagittal plane.


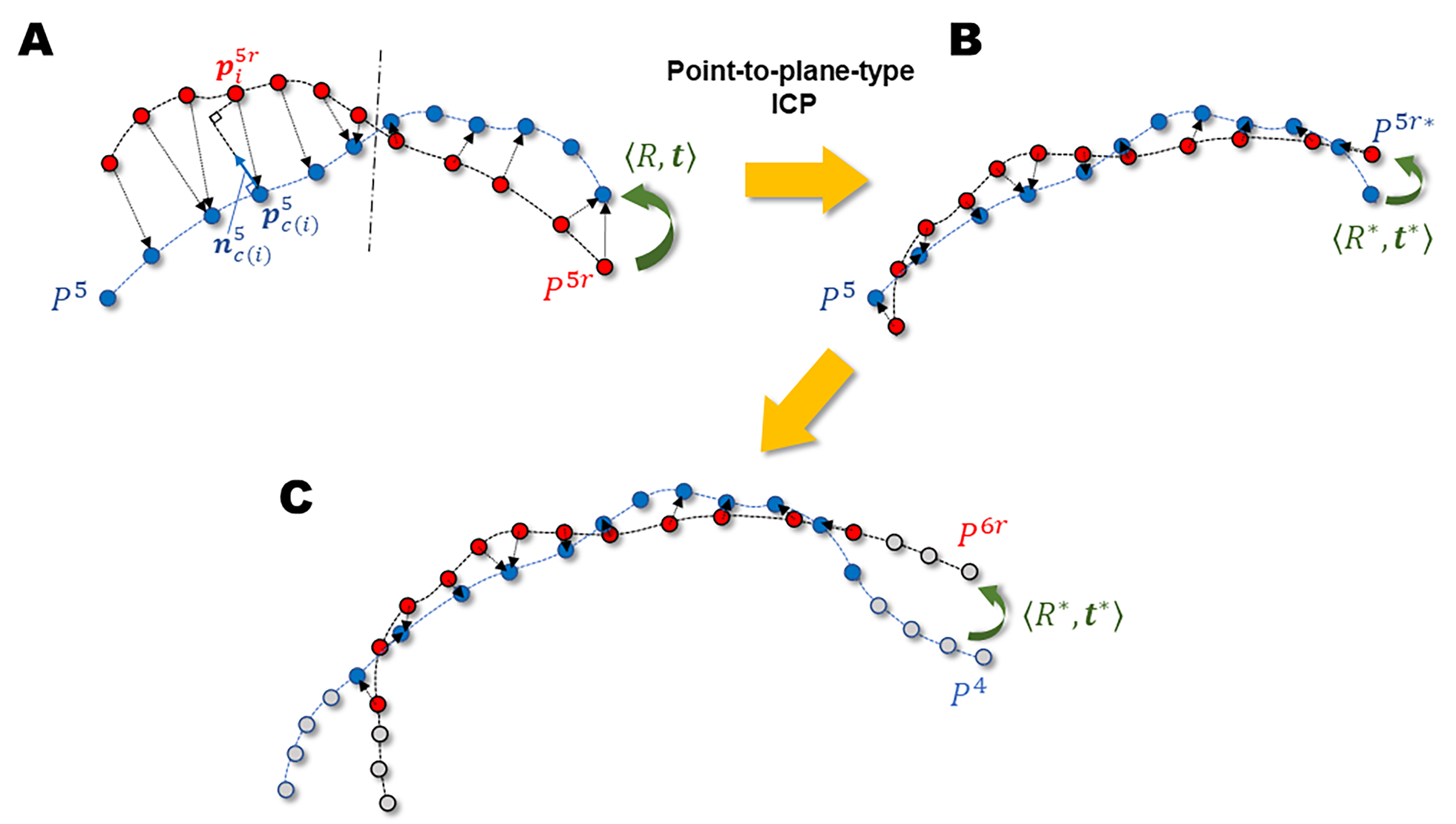


**Supplementary figure 3.** Finding the best fit between two point clouds using ICP. (A**)** Initial position and orientation of width-restricted point cloud
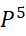
 and its reflected point cloud
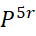
. **(B)** Point cloud
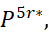
 which best fits
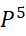
 under optimum rotation and translation
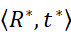
. **(C)** Optimum rotation and translation
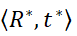
is applied to point cloud
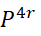
to provide the optimal reflected point cloud
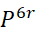
, which is fitted to the original point cloud
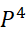
.


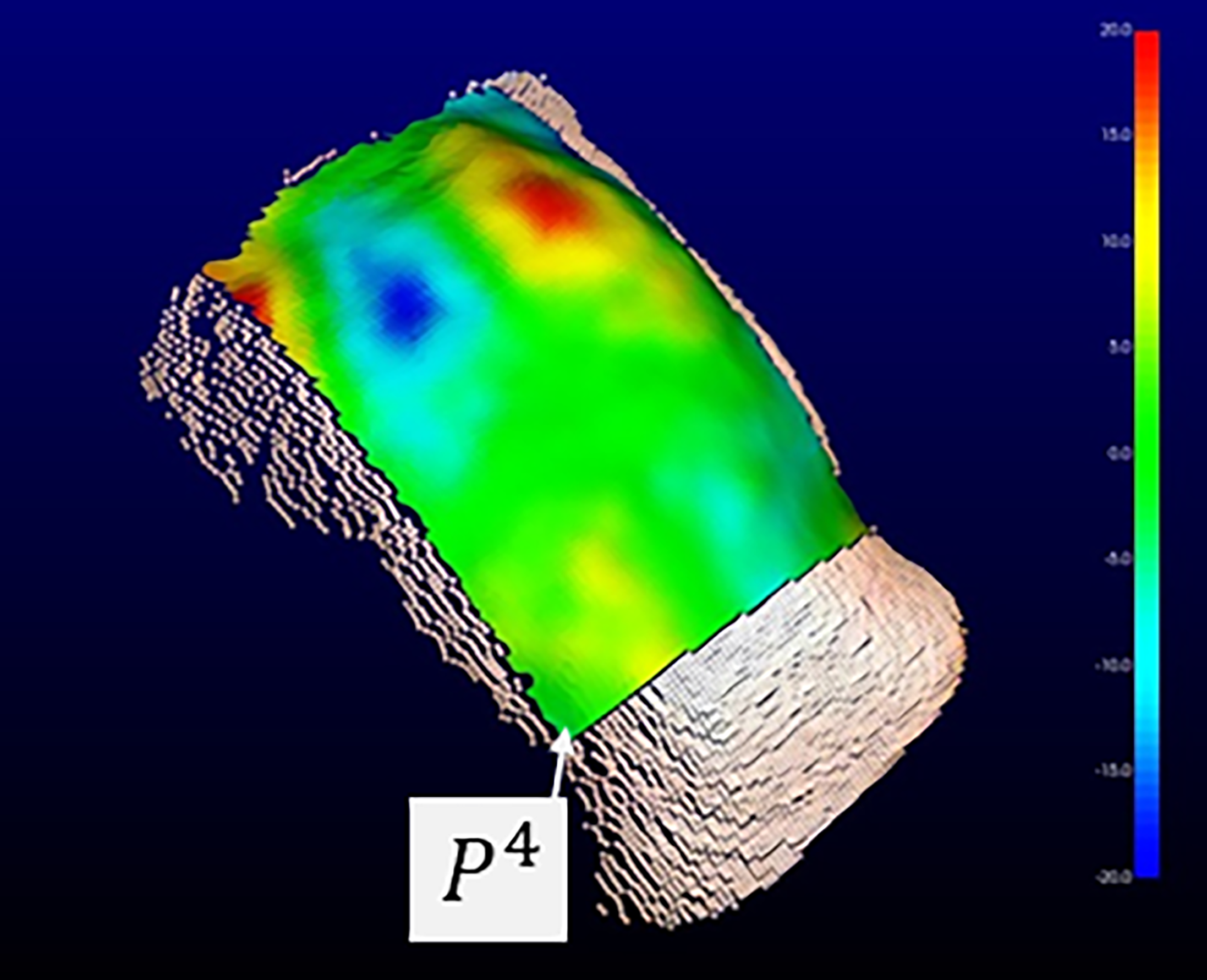


**Supplementary figure 4.** Colormap rendering of the deviation distribution
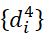
 between
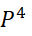
and
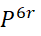
displayed on
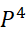
.


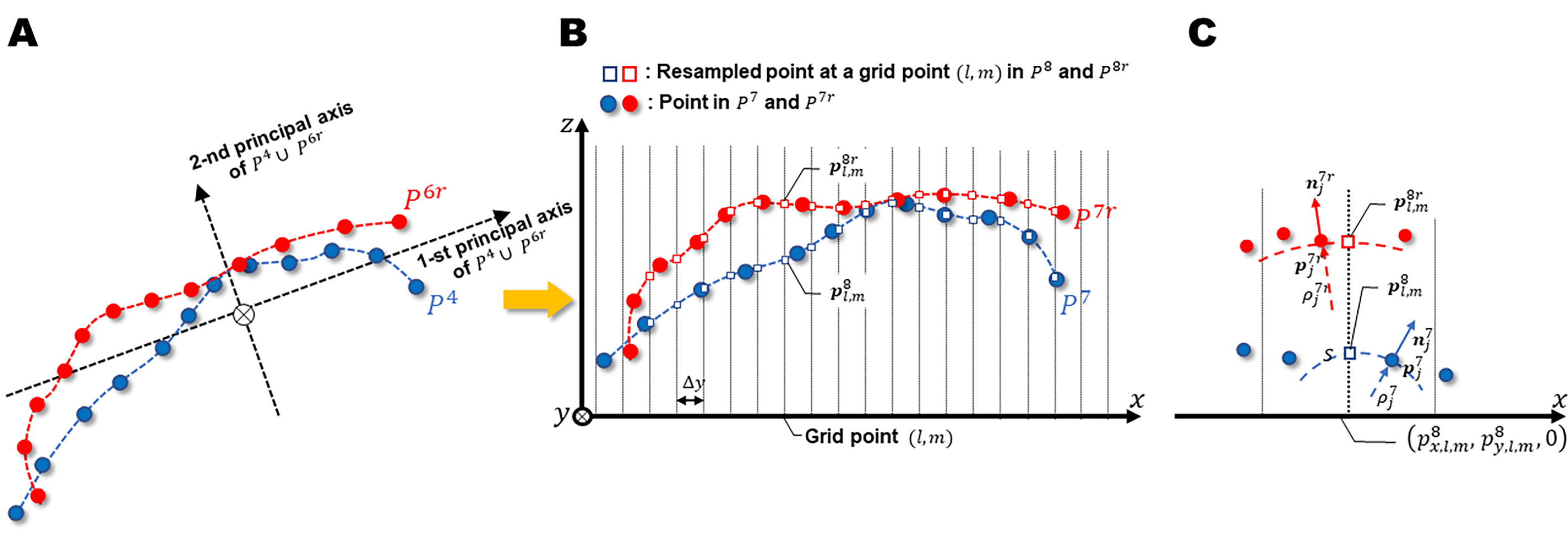


**Supplementary figure 5.** Precise extraction of deviations and estimation of the asymmetry index. **(A)** The orientation of
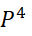
 and
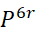
. **(B)** Resampling of point clouds
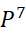
 and
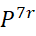
 at regular grid points. **(C)** Calculation of the resampled points
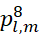
 and
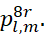


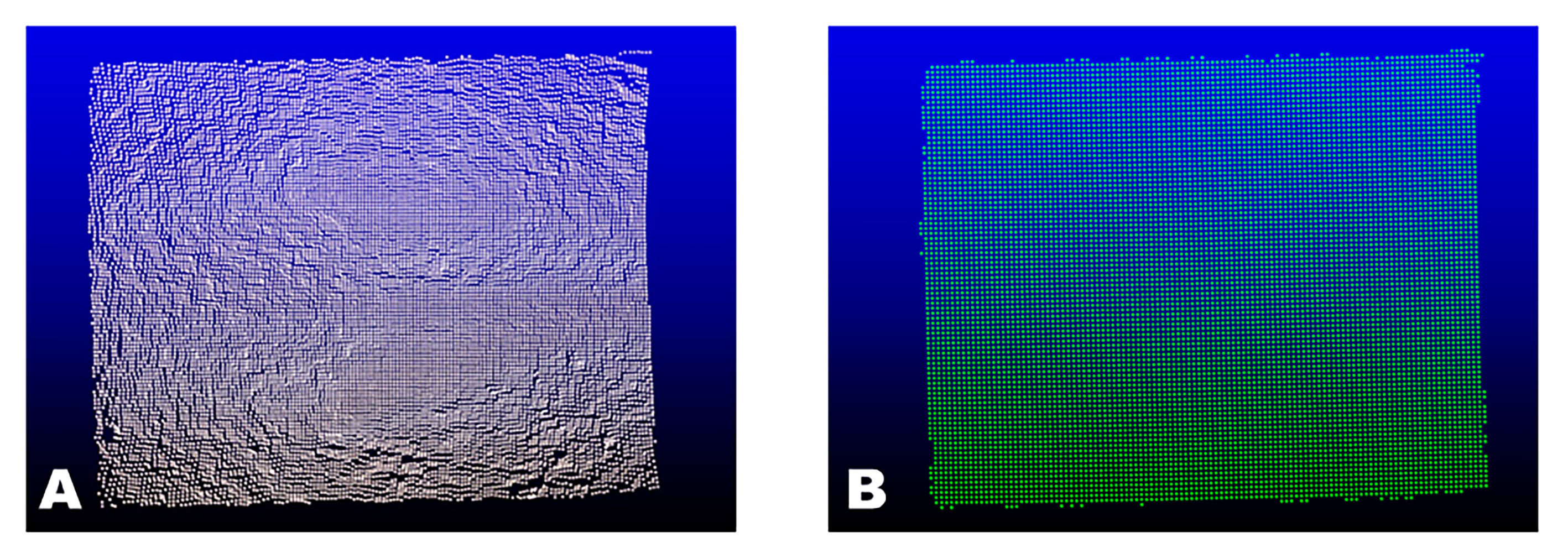


**Supplementary figure 6.** Points for the asymmetry index evaluation. **(A)** Point cloud
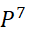
for asymmetry index evaluation before resampling. **(B)** The set of effective resampled points
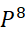
 at effective grid points for asymmetry index evaluation after resampling.


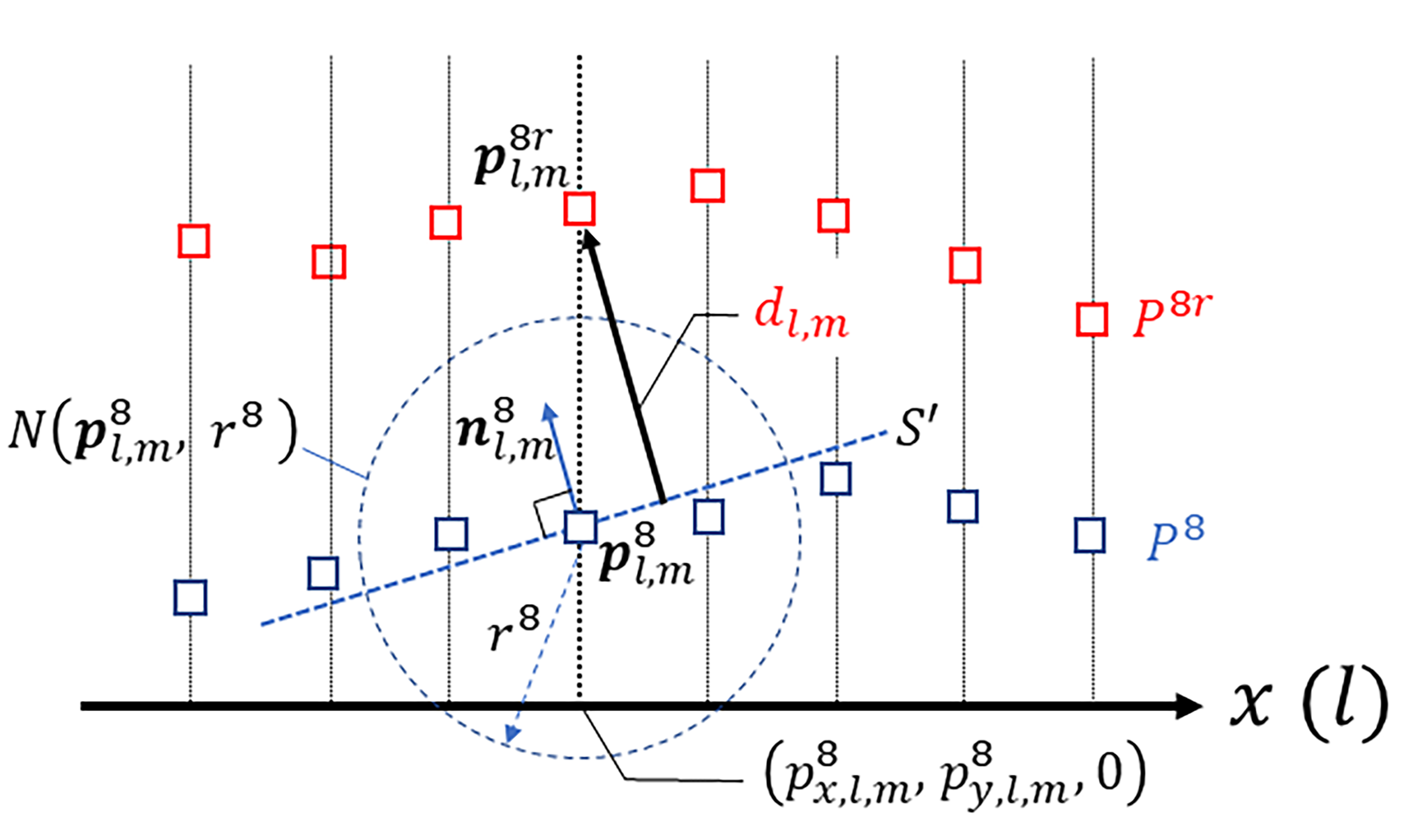


**Supplementary figure 7.** A new normal vector is estimated using principal component analysis of the neighboring point set.


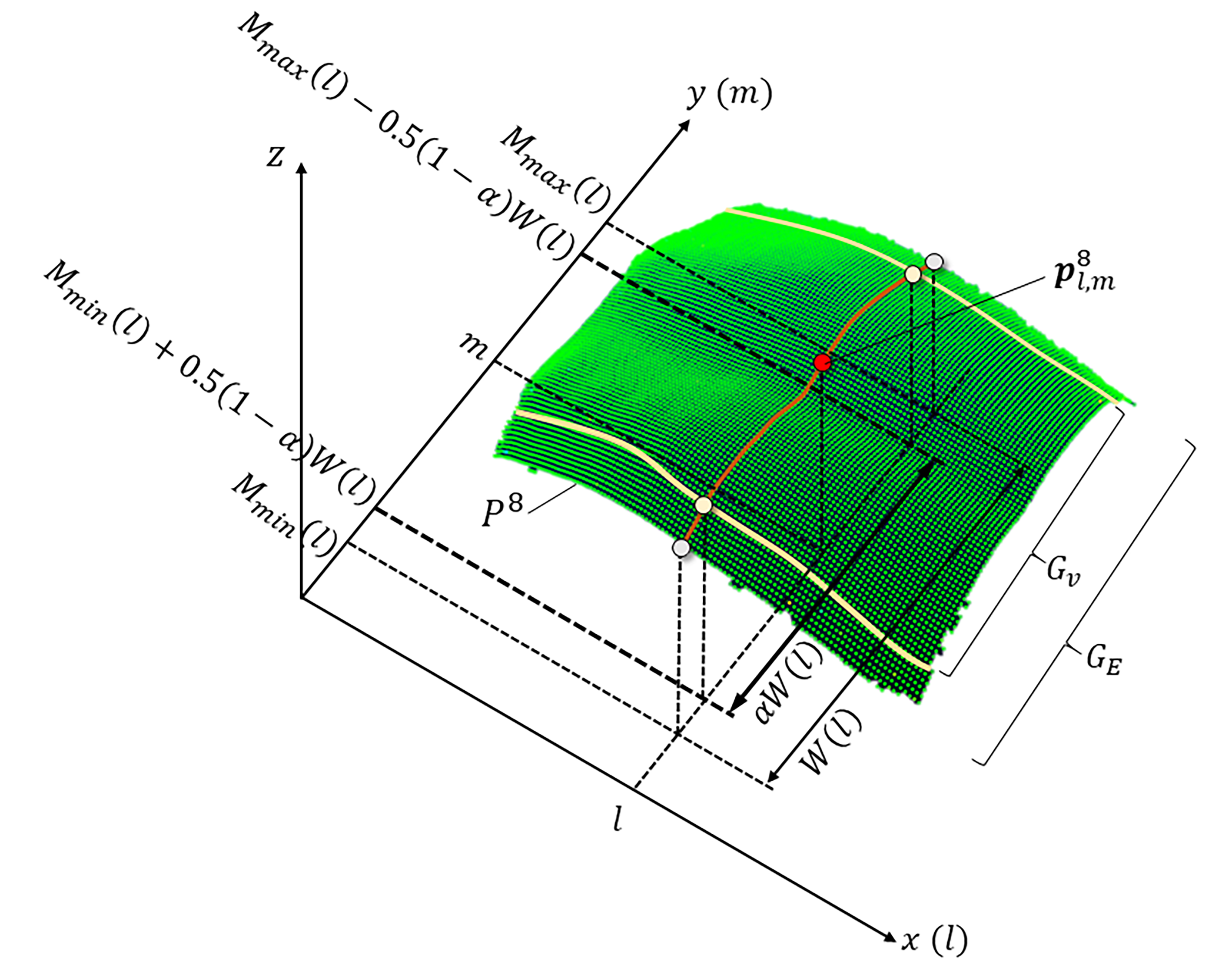


**Supplementary figure 8.** Grid point
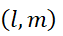
 is adopted as valid only if it lies in a valid range, such as
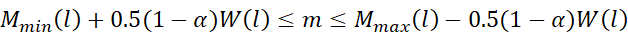
, where
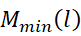
 and
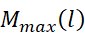
 are the minimum and maximum values of the *y* coordinate of the grid points at
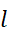
 included in
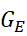
,
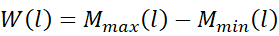
, and
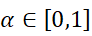
 is a parameter controlling the valid range.
